# Effect of free fatty acids on insulin secretion, insulin sensitivity and incretin effect – a narrative review

**DOI:** 10.20945/2359-3997000000313

**Published:** 2020-12-15

**Authors:** Valeria Bahdur Chueire, Elza Muscelli

**Affiliations:** 1 Universidade Católica de Campinas Hospital da Pontifícia Departamento de Endocrinologia Campinas SP Brasil Departamento de Endocrinologia, Hospital da Pontifícia Universidade Católica de Campinas, Campinas, SP, Brasil; 2 Universidade Estadual de Campinas Faculdade de Ciências Médicas Departamento de Clínica Médica Campinas SP Brasil Departamento de Clínica Médica, Faculdade de Ciências Médicas, Universidade Estadual de Campinas, Campinas, SP, Brasil

**Keywords:** FFAs, T2D, insulin secretion, incretin effect, insulin sensitivity

## Abstract

Deleterious effects of free fatty acids, FFAs, on insulin sensitivity are observed *in vivo* studies in humans. Mechanisms include impaired insulin signaling, oxidative stress, inflammation, and mitochondrial dysfunction, but the effects on insulin secretion are less well known. Our aim was to review the relationship of increased FFAs with insulin resistance, secretion and mainly with the incretin effect in humans. Narrative review. Increased endogenous or administered FFAs induce insulin resistance. FFAs effects on insulin secretion are debatable; inhibition and stimulation have been reported, depending on the type and duration of lipids exposition and the study subjects. Chronically elevated FFAs seem to decrease insulin biosynthesis, glucose-stimulated insulin secretion and β-cell glucose sensitivity. Lipids infusion decreases the response to incretins with unchanged incretin levels in volunteers with normal glucose tolerance. In contrast, FFAs reduction by acipimox did not restore the incretin effect in type-2 diabetes, probably due to the dysfunctional β-cell. Possible mechanisms of FFAs excess on incretin effect include reduction of the expression and levels of GLP-1 (glucagon like peptide-1) receptor, reduction of connexin-36 expression thus the coordinated secretory activity in response to GLP-1, and GIP (glucose-dependent insulinotropic polypeptide) receptors downregulation in islets cells. Increased circulating FFAs impair insulin sensitivity. Effects on insulin secretion are complex and controversial. Deleterious effects on the incretin-induced potentiation of insulin secretion were reported. More investigation is needed to better understand the extent and mechanisms of β-cell impairment and insulin resistance induced by increased FFAs and how to prevent them.

## INTRODUCTION

The high incidence of type 2 diabetes, T2D, in western countries has been attributed to the obesity epidemic and physical inactivity. A 114% increase of T2D prevalence is estimated until 2030, two thirds of which will be in developing countries. The International Diabetes Federation calculated 425 millions persons with diabetes worldwide in 2017 demonstrating the utmost importance of understanding the diabetes pathophysiology (1).

Type 2 diabetes is characterized mainly by defects in insulin action, β-cell dysfunction and chronic inflammation. The β-cell dysfunction is the major defect in most cases, but β-cell mass reduction alone is less likely to be the primary cause. A host of inherited genes, their environmental interaction and epigenetic mechanisms make tissues resistant to insulin and/or impairs insulin secretion. Clearly, insulin resistance is an important factor and has been reported in the liver, muscle and adipose tissue. It is found even in T2D first-degree relatives before obesity and hyperglycemia (2) suggesting a causal role. Type 2 diabetes manifests itself when β-cell becomes unable to adapt to chronic metabolic stress. Previously an “ominous octet” has been implicated in the pathophysiology of T2D: decreased insulin secretion, decreased incretin effect, increased glucagon secretion, increased hepatic glucose production, increased lipolysis, decreased muscle glucose uptake, increased renal glucose reabsorption, and brain neurotransmission dysfunction inducing local insulin resistance (3). Many other factors may contribute or may be added to this octet. In fact, activation of inflammatory pathways and impaired insulin mediated vasodilatation are associated to higher risk of T2D and contribute to muscle insulin resistance (4). The gut microbiota is involved with chronic inflammation and energy utilization from the diet. The type of consumed fat, such as saturated fatty acids (SFA) – in particular palmitic acid – changes the composition of the microbiota, that in turn, has deleterious effects on the end products from the gut bacterial fermentation. The increased circulating bacterial endotoxin contributes to inflammation in both obesity and diabetes. This is suggested as an additional factor for insulin resistance and impaired insulin secretion (4,5).

Another contributing factor is the elevated total plasma FFAs frequently observed in insulin resistance conditions (5). Of note, plasma FFAs concentrations depend on FFAs intake, de novo FFAs synthesis, triacylglycerol storage and lipolysis. Adipose tissue resistance to the antilipolytic insulin effect is frequent in T2D, impaired glucose tolerance, and centripetal obesity. This condition contributes to adipose tissue high lipid turnover, thereby increases plasma FFAs availability and concentration at fasting and after glucose or mixed meal ingestion (6). Consequently, elevated FFAs impair insulin action and secretion (5).

Our aim was to review the relationship between increased FFAs, insulin resistance and secretion. The FFAs induced metabolic changes called “lipotoxicity”, has been investigated more on insulin action than on insulin secretion and, the insulin secretion impairment is a major etiological factor for diabetes. Therefore, we revised especially their influence on insulin secretion and on the incretin effect in healthy and T2D individuals. A few animal studies were included to explain some hypotheses and mechanisms.

## FFAS EFFECTS ON INSULIN SENSITIVITY AND ON INSULIN SECRETION

Increased plasma FFAs impair glucose uptake and glycogen synthesis and stimulates hepatic gluconeogenesis in healthy people as well as in diabetes and obesity (6–8). Increased endogenous glucose production in response to FFAs infusion was also demonstrated in patients with type 1 diabetes, in whom there is no compensatory insulin secretion to reduce it (9). Elevated FFAs availability has been associated to lower insulin clearance by the liver, a common and early feature of insulin resistant states. However, this possibility has been debated, since increased glucose intake seems to impair insulin clearance more than FFAs (10). The FFAs-induced impairment of insulin sensitivity is observed in normoglycemia as well as in hyperglycemia (8). It has been shown that an overnight decrease of FFAs improves the insulin resistance (assessed by the hyperinsulinemic euglycemic clamp) and the hyperinsulinemia characteristic of people with obesity, impaired glucose tolerance and diabetes (11).

At cellular level, FFAs bind to cell membrane receptors of the GPR (G-protein coupled receptor, also known as free fatty acid receptor – FFA1) family in adipocytes, brain, pancreatic β-cells, enteroendocrine and immune cells. The CD36 fatty acid translocase protein, another membrane receptor FFAs binding, is expressed in myocytes, macrophages, endothelial cells, platelets, and enterocytes. Inside the cell FFAs must be converted to triacylglycerol for storage or undergo β-oxidation to be used as fuel by mitochondria. Increased FFAs impair triacylglycerol conversion and β-oxidation generating toxic lipids (diacylglycerol and ceramides), which cause oxidative and endoplasmic reticulum stress, mitochondrial dysfunction, and generation of reactive oxygen species (ROS) (12–15). Therefore, it induces inflammation, mostly in skeletal muscle and adipocytes but also in β-cells. Additionally, it has been described in the endothelial cells, providing a link to atherosclerotic vascular disease (7). Ceramides and diacylglycerol are associated with activation of TNFα and many stress-related kinases such as NFKbeta, P38MapKinase, JNK (c-Jun N-terminal kinases) and atypical PKC (protein kinase-C) isoforms in skeletal muscle. These enzymes decrease the insulin-signaling pathway by inducing serine/threonine phosphorylation of IRS1 (insulin receptor substrate 1). Thus, activation of the insulin signaling cascade, glucose uptake and its metabolism are all impaired. Reactive nitrogen species (RNS) also increase IRS1 nitration and degradation (14–17). Inside the cell, FFAs bind PPARs (peroxisome proliferator-activated receptors), which are ligand-activated nuclear transcription factors. They regulate FFAs uptake, storage and oxidation, so regulate glucose homeostasis indirectly. PPARγ plays a critical role linking unsaturated FFAs metabolism and inflammation reduction. Hence, FFAs metabolism may result in pro or anti-inflammatory signaling. Some long chain polyunsaturated FFAs (PUFA) are precursors of inflammation relief, others are precursors of pro and anti-inflammatory agents, stimulating both events (5). So, in this case, inflammation is a consequence of lipotoxicity (5,15). Saturated fatty acids, SFAs, activate toll-like receptor-4 (TLR-4), whose signaling pathway stimulates pro-inflammatory cytokines (IL-1β, IL-6, TNF-α). Besides SFAs activate the inflammasome multi-protein complex that induces mitochondrial dysfunction in many tissues by changing their Ca^++^ balance and also stimulate macrophages infiltration in the pancreatic β-cells. In contrast, several PUFA inhibit inflammasome and TLR-4 activation (5). Another possible mechanism for the insulin secretion impairment is a down regulation of the medium and long chain FFAs receptors (GPR40) in the β-cell, as observed in hyperlipidemic animal models before the diabetes development (18). In brief, the FFAs excess induces insulin resistance and β-cell dysfunction by many mechanisms, including a toxic inhibitory effect that ultimately induces β-cell apoptosis (19,20).

Plasma FFAs reduction by acipimox (a potent lipolysis inhibitor) improves insulin sensitivity and skeletal muscle mitochondrial adenosine triphosphate (ATP) synthesis in both insulin resistant obese with normal glucose tolerance and in patients with T2D, suggesting that the mitochondrial defect may be reversible (21,22). On the contrary, a 16 hour lipid infusion in T2D patients worsens insulin resistance (23).

Regarding insulin secretion, several studies have shown in humans that fatty acids, beyond glucose, are important for insulin secretion control (24). An acute and physiological increase of circulating FFAs sharply improves insulin secretion in animals and in healthy humans even if it stimulates gluconeogenesis (20,25). The acute FFAs-induced insulin secretion can occur by a direct effect, since long chain fatty acids infusion directly into the pancreatic artery of dogs increases insulin secretion (25).

In healthy subjects, contradictory results have been reported after lipids infusion. A 10hour overnight infusion did not change basal or GSIS (glucose-stimulated insulin secretion) (26), while a 24-hour Intralipid (10% triglyceride emulsion) infusion inhibited the first phase insulin secretion during an intravenous glucose tolerance test (27). In normal young subjects with no family history of diabetes, a prolonged fasting induced physiological increase in FFAs deteriorating β-cell function (28). In contrast, also in non-diabetic subjects, a 48h Intralipid infusion strongly potentiated glucose-induced insulin secretion (29). An important role of lipotoxicity in people at high risk for diabetes is suggested by worsened β-cell function in response to the FFAs rise. In fact, a four-day Intralipid infusion in volunteers with a family history of diabetes decreased the first and second phases of insulin secretion during a hyperglycemic clamp and during a mixed meal test, while it increased in healthy subjects without family history of diabetes. Thus, lipotoxicity in predisposed individuals was reported to both intravenous and oral glucose (30). Impaired insulin secretion after 48 hours of lipid infusion associated with decreased insulin clearance was also observed in non-diabetic obese subjects. However, in this same study, an increase in insulin secretion without changes in its clearance, i.e. no further impairment, was observed in diabetic patients with established β-cell dysfunction (31). In individuals with normal glucose tolerance with and without a T2D family history, FFAs decrease induced by acipimox was associated with a better acute insulin response to intravenous glucose (28,32). Moreover in patients with diabetes, FFAs reduction by acipimox increased GSIS over a previous improvement by dapagliflozin during an oral glucose tolerance test, OGTT (22).

Other deleterious effect of higher circulating FFAs is stimulation of VLDL and secretion of chylomicrons, potential atherogenic intestinally derived lipoprotein particles (33). Furthermore, increased FFAs plasma concentrations could inhibit GLP-1 (glucagon like peptide-1) secretion and/or its insulinotropic action as revised below.

## INCRETIN EFFECT AND INCRETINS

The Incretin Effect, characterized by the release of substances from the intestinal mucosa following nutrient ingestion (34,35), is responsible for approximately 50%-70% of the insulin response to oral glucose (36), thus the incretin effect has an important role in β-cell function in normal people. Reduced incretin effect seems to be linked to the pathophysiology of T2D or on the contrary, the reduction might be secondary to the diabetic state itself (37–39). The main incretin hormones are GIP (glucose-dependent insulinotropic polypeptide) and GLP-1, largely released by glucose, but also by proteins and lipids ingestion. The response of GIP is greater to proteins than to lipids in isocaloric solutions (40). As glucose, lipid and protein intake triggers a greater insulin secretion than their intravenous administration (41).

A 42-amino acid peptide, GIP is secreted by mucosa-specific K cells in the small intestine especially in the duodenum (36). Its receptors are expressed in the intestines, pancreatic islets, adipose tissue, heart, adrenal cortex, pituitary, and many brain regions (42). However their role is not well known in most of these sites.

Glucagon like peptide-1, a glucagon gene product is expressed in pancreatic α cells and in the mucosal L cells, one of the most abundant intestinal endocrine cells (36,43). These cells have been identified in the duodenum and jejunum, but they are found in greater number in the ileum, colon and also in the rectum (36,41,44). Secretion of GLP-1, one of the most potent stimulator of insulin-release begins minutes after nutrient ingestion, in amounts related to the food kind (45,46). The early secretion must be due to the transmission of signals from the proximal to the distal intestine via the autonomic and enteric nervous system (44,47) and also via gastrointestinal hormones and neuropeptides, such as substance P and gastrin-releasing peptide (GRP) (44). The release of GLP-1 in the proximal intestine must be by a subgroup of cells co-expressing GIP and the pro-glucagon gene (48). At physiological levels GIP might indirectly via the afferent vagal, or directly, at very high levels, interfere with the GLP-1 release. The efferent pathway must be the celiac branch of the vagus nerve, under the influence of GRP. At fasting and after oral glucose stimulation there is a pulsatile GLP-1 release that is inhibited by atropine (44), confirming the participation of the autonomic nervous system.

Glucagon like peptide-1 has effects other than to stimulate insulin secretion *in vivo*, such as inhibition of gastrointestinal motility, glucagon secretion, appetite and food intake; suppression of inflammation; promotion of mucosal integrity; vasodilation; natriuresis and somatostatin increase (36,37,49–51). Glucose-stimulated insulin secretion is increased by GLP-1 through improved glucose sensitivity (37) increased insulin biosynthesis and proinsulin and expression of other genes essentials for β-cell function such as glucokinase and GLUT-2 (37,52). It promotes differentiation of ductal progenitor cells into β-cells (53) and inhibits β-cell apoptosis (54–56). The hormone GIP also has extra incretin effects: it increases somatostatin and glucagon secretion, and lipoprotein lipase activity in adipose tissue, and induces body weight gain and bone formation.

## INCRETIN EFFECT AND INCRETIN CONCENTRATION IN T2D

The GLP-1 is three to five times more potent than GIP in T2D, since these patients are resistant to the GIP effect on insulin secretion (41,57). In subjects with normal glucose tolerance, GIP seems to be more important than GLP-1 to mediate the incretin effect (58), but a similar potency has also been reported (36). Contradictory results regarding GLP-1 concentrations have been described in T2D. More frequently it seems to be normal with a partially impaired effect while GIP secretion is usually normal or increased (36,37,59–61). A cross-sectional study showed a slight decrease in GLP-1 release after a mixed meal in volunteers with impaired glucose tolerance, and a more severe reduction in T2D patients (60), suggesting a parallel loss of GLP-1 secretion as diabetes progresses. The GIP response to an OGTT associated with elevated circulating FFAs was higher in non-diabetic obese women compared to lean. Hence, GIP may assume greater importance to maintain insulin secretion in obese individuals who have decreased GLP-1 secretion, preventing hyperglycemia (62). Many factors may be implicated in the reported decrease of GLP-1 secretion in T2D (37). As a glucagonostatic hormone, GLP-1 inhibits glucagon secretion (51,57), which is paradoxically increased in diabetes. Increased plasma glucagon at fasting and post glucose stimulus starts before glucose intolerance and diabetes as a consequence of insulin resistance in α-cells (63–65). Glucagon, in turn, suppresses GLP-1 (44), perhaps through a direct effect on L-cell receptors. Furthermore, diabetic patients often have slow antro-duodenal nutrient transit, as well as gastrointestinal autonomic neuropathy (44,64), delaying L cells stimulation. A direct effect of FFAs is possible, since in rat insulinoma INS-1E cells and in isolated islets of db/db mice (diabetic and obese model), palmitate decreased the expression and levels of the GLP-1 receptor and impaired insulin secretion (66).

In patients with T2D, the strong β-cell resistance to GIP and a slight resistance to GLP-1 could be secondary to glucotoxicity (57) due to a down regulation of their receptors induced by hyperglycemia (37,67). In turn, decreased incretin receptor signaling seems to contribute to gluco-lipotoxicity in combination with other pathways involving the endoplasmic reticulum and oxidative stress (68). Thus, β-cell of diabetic patients might not express GIP receptors or they are defective (66,67). However, these defects may be explained by β-cells reduction or dysfunction, more likely by the combination of both possibilities (69,70). Little information is available on the effect of FFAs on incretin-stimulated insulin secretion in humans, despite recent evidence in human islets that it might interfere with incretin function (71).

Incretin effect is decreased in T2D, as well as in other insulin resistance states, such as obesity and glucose intolerance (59,63,72–74). The underlying mechanisms of the incretin effect impairment are not completely elucidated. According to some studies, it happens as a consequence of the “diabetic state”, not being an etiological factor, since it improves after diabetes compensation (37–39). Alternatively it may be genetically based (36,75). Some authors give more importance to the reduced insulinotropic GIP effect than to the decreased GLP-1 concentrations to impair the incretin effect in diabetes (37,57,58). As discussed below one of the mechanisms implicated in the impaired incretin effect in diabetes might be the FFAs excess.

## INFLUENCE OF FFAS EXCESS ON THE INCRETIN EFFECT

Human studies to verify the consequences of FFAs excess on GLP-1 response and its ability to potentiate β-cell function are of utmost importance as well as the understanding of insulin secretion and the factors that modify it, *in vivo* and in humans. As long as one of the incretins effects is the potentiation of insulin secretion, we investigated in volunteers with normal glucose tolerance, whether incretin-induced insulin secretion is reduced by an acute elevation of plasma lipids, and if an acute reduction of them is able to improve the incretin effect in patients with T2D (76). We evaluated many insulin secretion parameters from an OGTT and from a corresponding isoglycemic intravenous glucose infusion test using a mathematical model (77): total insulin secretion during both tests, glucose-induced and incretin-induced potentiation of insulin secretion and β-cell glucose sensitivity (β-CGS). The last one is evaluated by a dose-response curve of insulin secretion to glucose levels variation, and it is an important parameter of β-Cell function. In individuals with normal glucose tolerance, the acute FFAs increase caused a marked decrease in incretin-induced potentiation and a slight deterioration of insulin sensitivity and consequently of glucose tolerance, Because of this, it increased total and the first phase insulin secretion, without modifying β-CGS. The potentiation of incretin-dependent insulin secretion decreased even if the secretion of incretins (plasma concentrations) were unchanged. Conversely, in the T2D patients, administration of acipimox caused a significant reduction in FFAs, glycemia and insulinemia, as well as improving insulin sensitivity. However, β-CGS and both, potentiation of glucose-dependent insulin secretion and incretin-induced potentiation were not improved. Hence, the impact of FFAs on the incretin effect is essentially dependent on the β-cell function, i.e., their increase may impair the incretin effect in a normal β-cell. However, FFAs reduction is not able to restore the incretin effect in a dysfunctional β-cell, at least under these conditions. These findings demonstrate that FFAs influence the incretin effect in healthy humans (76).

The impairment of incretin function, caused by FFAs exposure, as we demonstrated in volunteers with normal glucose tolerance may be due to several mechanisms. One possibility is the reduction of the expression and levels of the GLP-1 receptor as observed in rat insulinoma INS-1E cells and in isolated islets of db/db mice. This reduction was associated to a deficiency of cAMP production, protein phosphorylation of cAMP-responsive elements binding protein (p-CREB) and insulin secretion (66). Furthermore, GLP-1 is responsible for the recruitment of a β-cell network essential to synchronize rapid increases in glucose-induced insulin secretion. Gap junctions in human β-cells, through connexin 36 (Cx36), are important for incretin-stimulated insulin secretion (71). In human islets addition of palmitate reduced Cx36 expression and the coordinated secretory activity in response to GLP-1 and GIP, and consequently reduced insulin release. Therefore, raised FFAs might disrupt the GLP-1-sensitive syncytium. The association of hyperglycemia and higher FFAs concentration frequently found in patients with diabetes seems to be synergistically toxic to islets (15), where it down regulates GPR40 expression in all cell types (18). Insulin secretion impairment was observed also in human islets exposed to oleate or palmitate for 48h (78). In a recent speculative study in T2D individuals, the clinical efficacy of liraglutide, a GLP-1 receptor agonist, was related to the baseline plasma triglycerides and C-peptide levels (79), emphasizing the importance of lipid control.

**Figure 1:**
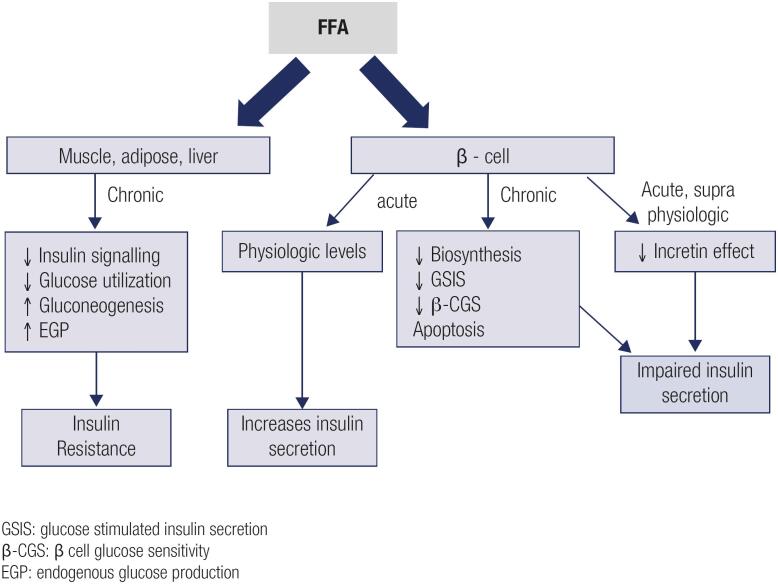
FFAs effects on insulin sensitivity and insulin secretion – possible mechanisms.

In brief, acute physiological FFAs increase stimulates insulin secretion. Chronic elevations decrease insulin biosynthesis, GSIS, β-CGS and induce apoptosis. Furthermore, acute supra physiological levels impair the incretin effect (Figure 1).

In conclusion, FFAs impairment of insulin sensitivity has been repeatedly reported. However, the effects and mechanisms of increased circulating FFAs on insulin secretion are not well known. Most *in vivo* and *in vitro* studies suggest deleterious effects on the incretin-induced potentiation of insulin secretion involving β-cell toxicity, decreased incretin receptors on the β-cells, and disrupted GLP-1-sensitive syncytium, and even effects on the therapeutic response to GLP-1 agonists. The impairment of insulin secretion FFAs-induced is observed even in volunteers with normal glucose tolerance, but FFAs reduction does not restore insulin secretion in subjects with dysfunctional β-cell. Furthermore, even in youth with normoglycemia and obesity, decreased muscle, adipose and hepatic insulin sensitivity was inversely associated to FFAs and liver fat (80). In this way, more investigation is necessary to understand which interventions and when reductions of plasma lipids are more efficacious to preserve β-cell function and insulin action.

## References

[1] International Diabetes Federation: IDF Atlas (2017). https://www.idf.org/e-library/epidemiology-research/diabetes-atlas.html.

[2] Cusi K (2009). Lessons learned from studying families genetically predisposed to type 2 diabetes mellitus. Curr Diab Rep.

[3] DeFronzo RA (2009). From the triumvirate to the ominous octet: a new paradigm for the treatment of type 2 diabetes mellitus– Banting Lecture. Diabetes.

[4] DeFronzo RA, Ferrannini E, Groop L, Henry RR, Herman WH, Holst JJ (2015). Type 2 diabetes mellitus. Nat Rev Dis Primers.

[5] Soboczak AIS, Blindauer CA, Stewart AJ (2019). Changes in plasma free fatty acids associated with type-2 diabetes. Nutrients.

[6] Gastaldelli A, Gaggini M, DeFronzo RA (2017). Role of adipose tissue insulin resistance in the natural history of type 2 diabetes: results from the San Antonio Metabolism Study. Diabetes.

[7] Boden G (2008). Obesity and free fatty acids. Endocrinol Metab Clin North Am.

[8] Ferrannini E, Barret EJ, Bevilacqua S, DeFronzo RA (1983). Effect of fatty acids on glucose production and utilization in man. J Clin Invest.

[9] Staehr P, Hother-Nielsen O, Landau BR, Chandramouli V, Holst JJ, Beck-Nielsen H (2003). Effects of free fatty acids per se on glucose production, gluconeogenesis and glycogenolysis. Diabetes.

[10] Bojsen-Møller KN, Lundsgaard AM, Madsbad S, Kiens B, Holst JJ (2018). Hepatic Insulin Clearance in Regulation of Systemic Insulin Concentrations – Role of Carbohydrate and Energy Availability. Diabetes.

[11] Santomauro AT, Boden G, Silva ME, Rocha DM, Santos RF, Ursich MJ (1999). Overnight lowering of free fatty acids with Acipimox improves insulin resistance and glucose tolerance in obese diabetic and nondiabetic subjects. Diabetes.

[12] Koves TR, Ussher JR, Noland RC, Slentz D, Mosedale M, Ilkayeva O (2008). Mitochondrial overload and incomplete fatty acid oxidation contribute to skeletal muscle insulin resistance. Cell Metab.

[13] Capurso C, Capurso A (2012). From excess adiposity to insulin resistance: the role of free fatty acids. Vascul Pharmacol.

[14] Cusi K (2010). The role of adipose tissue and lipotoxicity in the pathogenesis of type 2 diabetes. Curr Diab Rep.

[15] Giacca A, Xiao C, Oprescu AI, Carpentier AC, Lewis GF (2011). Lipid-induced pancreatic β-cell dysfunction: focus on in vivo studies. Am J Physiol Endocrinol Metab.

[16] Yu C, Chen Y, Cline GW, Zhang D, Zong H, Wang Y (2002). Mechanism by which fatty acids inhibit activation of insulin receptor substrate-1 (IRS-1) – associated phosphatidylinositol 3-kinase activity in muscle. J Biol Chem.

[17] Kruszynska YT, Worrall DS, Ofrecio J, Frias JP, Macaraeg G, Olefsky JM (2002). Fatty acid-induced insulin resistance: decreased muscle PI3K activation but unchanged Akt phosphorylation. J Clin Endocrinol Metab.

[18] Abaraviciene SM, Muhammed SJ, Amisten S, Lundquist I, Salehi A (2013). GPR40 protein levels are crucial to the regulation of stimulated hormone secretion in pancreatic islets. Lessons from spontaneous obesity-prone and non-obese type 2 diabetes in rats. Molec Cell Endocrinol.

[19] Lupi R, Del Prato S (2008). Beta-cell apoptosis in type 2 diabetes: quantitative and functional consequences. Diabetes Metab.

[20] McGarry JD, Dobbins RL (1999). Fatty acids, lipotoxicity and insulin secretion. Diabetologia.

[21] Daniele G, Eldor R, Merovci A, Clarke GD, Xiong J, Tripathy D (2014). Chronic reduction of plasma free fatty acid improves mitochondrial function and whole-body insulin sensitivity in obese and type 2 diabetic individuals. Diabetes.

[22] Merovci A, Abdul-Ghani M, Mari A, Solis-Herrera C, Xiong J, Daniele G (2016). Effect of dapagliflozin with and without acipimox on insulin sensitivity and insulin secretion in T2DM males. J Clin Endocrinol Metab.

[23] Carpentier A, Bourbonnais A, Frisch F, Giacca A, Lewis GF (2010). Plasma nonesterified fatty acid intolerance and hyperglycemia are associated with intravenous lipid-induced impairment of insulin sensitivity and disposition index. J Clin Endocrinol Metab.

[24] Kruszynska YT, Mulford MI, Yu JG, Armstrong DA, Olefsky JM (1997). Effects of non-esterified fatty acids on glucose metabolism after glucose ingestion. Diabetes.

[25] Crespin SR, Greenough WB, Steinberg D (1973). Stimulation of insulin secretion by long-chain free fatty acids. A direct pancreatic effect. J Clin Invest.

[26] Amery CM, Round RA, Smith JM, Nattrass M (2000). Elevation of plasma fatty acids by tem-hour intralipid infusion has no effect on basal or glucose-stimulated insulin secretion in normal man. Metabolism.

[27] Paolisso G, Gambardella A, Amato L, Tortoriello R, D’Amore A, Varricchio M (1995). Opposite effects of short and long-term fatty acid infusion on insulin secretion in healthy subjects. Diabetologia.

[28] Salgin B, Marcovecchio ML, Humphreys SM, Hill N, Chassin LJ, Lunn DJ (2009). Effects of prolonged fasting and sustained lipolysis on insulin secretion and insulin sensitivity in normal subjects. Am J Physiol Endocrinol Metab.

[29] Boden G, Chen X, Rosner J, Baron M (1995). Effects of a 48-hour fat infusion on insulin secretion and glucose utilization. Diabetes.

[30] Kashyap S, Belfort R, Gastaldelli A, Pratipanawatr T, Berria R, Pratipanawatr W (2003). A sustained increase in plasma free fatty acids impairs insulin secretion in nondiabetic subjects genetically predisposed to develop type 2 diabetes. Diabetes.

[31] Carpentier A, Mittelman SD, Bergman RN, Giacca A, Lewis GF (2000). Prolonged elevation of plasma free fatty acids impairs pancreatic beta-cell function in obese nondiabetic humans but not in individuals with type 2 diabetes. Diabetes.

[32] Paolisso G, Tagliamonte MR, Rizzo MR, Gualdiero P, Saccomanno F, Gambardella A (1998). Lowering fatty acids potentiates acute insulin response in first degree relatives of people with type II diabetes. Diabetologia.

[33] Xiao C, Dash S, Morgantini C, Lewis GF (2014). New and emerging regulators of intestinal lipoprotein secretion. Atherosclerosis.

[34] Perley MJ, Kipnis DM (1967). Plasma insulin responses to oral and intravenous glucose: studies in normal and diabetic subjects. J Clin Invest.

[35] Nauck MA, Homberger E, Siegel E, Allen R, Eaton RP, Ebert R (1986). Incretin effects of increasing glucose loads in man calculated from venous insulin and C-peptide responses. J Clin Endocrinol Metab.

[36] Vilsboll T, Holst JJ (2004). Incretins, insulin secretion and Type 2 diabetes mellitus. Diabetologia.

[37] Nauck MA, Meier JJ (2018). Incretin Hormones: Their role in health and disease. Diabetes Obes Metab.

[38] Meier JJ, Nauck MA, Sepmann N, Greulich M, Holst JJ, Deacon CF (2003). Similar insulin secretory response to a gastric inhibitory polypeptide bolus injection at euglycemia in first-degree relatives of patients with type 2 diabetes and control subjects. Metabolism.

[39] Knop FK, Vilsboll T, Hojberg PV, Larsen S, Madsbad S, Holst JJ (2007). The insulinotropic effect of GIP is impaired in patients with chronic pancreatitis and normal glucose tolerance. Regul Pept.

[40] Carr RD, Larsen MO, Winzell MS, Jelic K, Lindgren O, Deacon CF (2008). Incretin and islet hormonal responses to fat and protein ingestion in healthy men. Am J Physiol Endocrinol Metab.

[41] Holst JJ (2019). From the incretin concept and the discovery of GLP-1 to today's diabetes therapy. Front Endocrinol (Lausanne).

[42] Usdin TB, Mezey E, Button DC, Brownstein MJ, Bonner TI (1993). Gastric inhibitory polypeptide receptor, a member of the secretin-vasoactive intestinal peptide receptor Family, is widely distributed in peripheral organs and the brain. Endocrinology.

[43] Bell GI, Sanchez-Pescador R, Laybourn PJ, Najarian RC (1983). Exon duplication and divergence in the human pre-proglucagon gene. Nature.

[44] Nauck MA, Vardarli I, Deacon CF, Holst JJ, Meier JJ (2011). Secretion of GLP-1 in type 2 diabetes: what is up, what is down?. Diabetologia.

[45] Elliot RM, Morgan LM, Tredger JA, Deacon S, Wright J, Marks V (1993). Glucagon-like peptide-1(7-36) amide and glucose-dependent insulinotropic polypeptide secretion in response to nutrient ingestion in man: acute post-prandial and 24-h secretion patterns. J Endocrinol.

[46] Holst JJ (1994). Glucagon-like peptide 1: a newly discovered gastrointestinal hormone. Gastroenterology.

[47] Hermann-Rinke C, Vöge A, Hess M, Göke B (1995). Regulation of GLP-1 secretion from rat ileum by neuro-transmitters and peptides. J Endocrinol.

[48] Mortensen K, Christensen LL, Holst JJ, Orskov C (2003). GLP-1 and GIP are colocalized in a subset of endocrine cells in the small intestine. Regul Pept.

[49] Bang-Berthelsen CH, Holm TL, Pyke C, Simonsen L, Søkilde R, Pociot F (2016). GLP-1 induces barrier protective expression in Brunner's glands and regulates colonic inflammation. Inflamm Bowel Dis.

[50] Holst JJ, Vilsboll T, Deacon CF (2009). The incretin system and its role in type 2 diabetes mellitus. Mol Cell Endocrinol.

[51] Hare KJ, Vilsboll T, Asmar M, Deacon CF, Knop FK, Holst JJ (2010). The glucagonostatic and insulinotropic effects of GLP-1 contribute equally to its glucose-lowering action. Diabetes.

[52] Buteau J, Roduit R, Susini S, Prentki M (1999). GLP-1 promotes DNA synthesis, activates phosphatidylinositol 3-kinase and increases transcription factor pancreatic and duodenal homeobox gene1 (PDX-1) DNA binding activity in beta (INS-1)-cells. Diabetologia.

[53] Zhou J, Wang X, Pineyro MA, Egan JM (1999). Glucagon-like peptide 1 and exedin-4 convert pancreatic AR42J cells into glucagon- and insulin-producing cells. Diabetes.

[54] Li Y, Hansotia T, Yusta B, Ris F, Halban PA, Drucker DJ (2003). Glucagon-like peptide-1 receptor signaling modulates beta-cell apoptosis. J Biol Chem.

[55] Farilla L, Hui H, Bertolotto C, Kang E, Bulotta A, Di Mario U (2002). GLP-1 promotes islets cell growth and inhibits apoptosis in Zucker diabetic rats. Endocrinology.

[56] Fan R, Li X, Gu X, Chan JC, Xu G (2010). Exendin-4 protects pancreatic beta cells from human islet amyloid polypeptide-induced cell damage: potential involvement of AKT and mitochondria biogenesis. Diabetes Obes Metab.

[57] Nauck MA, Heimesaat MM, Orskov C, Holst JJ, Ebert R, Creutzfeldt W (1993). Preserved incretin activity of GLP-1[7-36amide] but not of synthetic human GIP in patients with type 2 diabetes mellitus. J Clin Invest.

[58] Nauck MA, Bartels E, Orskov C, Ebert R, Creutzfeldt W (1993). Additive insulinotropic effects of exogenous synthetic human GIP and GLP-1(7-36) amide infused at near-physiological insulinotropic and glucose concentrations. J Clin Endocrinol Metab.

[59] Muscelli E, Mari A, Calsolaro A, Camastra S, Seghieri G, Gastaldelli A (2008). Separate impact of obesity and glucose tolerance on the incretin effect in normal subjects and type 2 diabetic patients. Diabetes.

[60] Toft-Nielsen MB, Damholt MB, Madsbad S, Hilsted LM, Hughes TE, Michelsen BK (2001). Determinants of the impaired secretion of GLP-1 in type 2 diabetic patients. J Clin Endocrinol Metab.

[61] Vaag AA, Holst JJ, Volund A, Beck-Nielsen H (1996). Gut incretin hormones in identical twins discordant for NIDDM – evidence for decrease GLP-1 secretion during oral glucose ingestion in NIDDM twins. Eur J Endocrinol.

[62] Ranganath L, Norris F, Morgan J, Wright J, Marks V (1999). The effect of circulating non-esterified fatty acids on the entero-insular axis. Eur J Clin Invest.

[63] Knop FK, Aaboe K, Vilsboll T, Volund A, Holst JJ, Krarup T (2012). Impaired incretin effect and fasting hyperglucagonaemia characterizing type 2 diabetic subjects are early signs of dysmetabolism in obesity. Diabetes Obes Metab.

[64] Borghi VC, Wajchenberg BL, Cesar PF (1984). Plasma glucagon suppressibility after oral glucose in obese subjects with normal and impaired glucose tolerance. Metabolism.

[65] Lefèbvre PJ (1995). Glucagon and its family revisited. Diabetes Care.

[66] Kang ZF, Deng Y, Zhou Y, Fan RR, Chan JC, Laybutt DR (2013). Pharmacological reduction of NEFA restores the efficacy of incretin-based therapies through GLP-1 receptor signaling in the beta cell in mouse models of diabetes. Diabetologia.

[67] Shu L, Matveyenko AV, Kerr-Conte J, Cho JH, McIntosh CH, Maedler K (2009). Decreased TCF7L2 protein levels in type 2 diabetes mellitus correlate with downregulation of GIP and GLP-1receptors and impaired beta-cell function. Hum Mol Genet.

[68] Poitout V, Robertson RP (2008). Glucolipotoxicity: fuel excess and beta-cell dysfunction. Endocr Rev.

[69] Kloppel G, Lohr M, Habich K, Oberholzer M, Heitz PU (1985). Islet pathology and the pathogenesis of type 1 and type 2 diabetes mellitus revisited. Surv Synth Pathol Res.

[70] Polonsky KS (1995). The B-cell in diabetes: from molecular genetics to clinical research. Diabetes.

[71] Hodson DJ, Mitchell RK, Bellomo LA, Sun G, Sun G, Vinet L (2013). Lipotoxicity disrupts incretin-regulated human β cell connectivity. J Clin Invest.

[72] Nauck M, Stockmann F, Ebert R, Creutzfeldt W (1986). Reduced incretin effect in type 2 diabetes. Diabetologia.

[73] Muscelli E, Mari A, Natali A, Astiarraga BD, Camastra S, Frascerra S (2006). Impact of incretin hormones on beta-cell function in subjects with normal or impaired glucose tolerance. Am J Physiol Endocrinol Metab.

[74] Muscelli E, Casolaro A, Gastaldelli A, Mari A, Seghieri G, Astiarraga B (2012). Mechanisms for the antihyperglycemic effect of sitagliptin in patients with type 2 diabetes. J Clin Endocrinol Metab.

[75] Meier JJ, Hucking K, Holst JJ, Deacon CF, Schmiegel WH, Nauck MA (2001). Reduced insulinotropic effect of gastric inhibitory polypeptide in fist-degree relatives of patients with type 2 diabetes. Diabetes.

[76] Astiarraga B, Chueire VB, Souza AL, Pereira-Moreira R, Monte Alegre S, Natali A (2018). Effects of acute NEFA manipulation on incretin-induced insulin secretion in participants with or whitout type 2 diabetes. Diabetologia.

[77] Tura A, Muscelli E, Gastaldelli A, Ferrannini E, Mari A (2014). Altered pattern of the incretin effect as assessed by modelling in individuals with glucose tolerance ranging from normal to diabetic. Diabetologia.

[78] Zhou YP, Grill V (1995). Long term exposure to fatty acids and ketones inhibits B-cell functions in human pancreatic islets of Langerhans. J Clin Endocrinol Metab.

[79] Tanabe A, Kaneto H, Kamei S, Hirukawa H, Shimoda M, Kimura T (2016). Clinical effects of liraglutide are possibly influenced by hypertriglyceridemia and remaining pancreatic β-cell function in subjects with type 2 diabetes mellitus. J Diabetes Complications.

[80] Cree-Green M, Wiromrat P, Stuppy JJ, Thurston J, Bergman BC, Baumgartner AD (2019). Muscle Insulin Resistance in Youth with Obesity and Normoglycemia is Associated with Altered Fat Metabolism. Obesity (Silver Spring).

